# Diversity and phylogenetic relationships of European species of *Crepidostomum* Braun, 1900 (Trematoda: Allocreadiidae) based on rDNA, with special reference to *Crepidostomum oschmarini* Zhokhov & Pugacheva, 1998

**DOI:** 10.1186/s13071-018-3095-y

**Published:** 2018-09-28

**Authors:** Romualda Petkevičiūtė, Virmantas Stunžėnas, Alexander E. Zhokhov, Larisa G. Poddubnaya, Gražina Stanevičiūtė

**Affiliations:** 1 Institute of Ecology of Nature Research Centre, Akademijos str. 2, LT-08412 Vilnius, Lithuania; 2 Papanin Institute for Biology of Inland Waters, Russian Academy of Sciences, Borok, Russia

**Keywords:** *Crepidostomum oschmarini*, ITS2 rDNA, *28S*, Molecular phylogeny, Life-cycles, Tegumental topography, Morphology, Stone loach *Barbatula barbatula*, European bullhead *Cottus gobio*

## Abstract

**Background:**

Within the genus *Crepidostomum* Braun, 1900, identification of species and taxonomic decisions made only on the basis of adult morphology have resulted in great problems associated with evaluating actual diversity and validity of species. Life-cycle data, while equal in importance to adult characters, are scarce, controversial or incomplete for most *Crepidostomum* spp. In this study, rDNA sequences generated from adult and larval *Crepidostomum* spp. and some other allocreadiid species were analysed to reveal the diversity and phylogenetic relationships of the species and their host range. Detailed morphological description based on light microscopy, SEM tegumental surface topography and genetic data are provided for the poorly known trematode *C. oschmarini* Zhokhov & Pugacheva, 1998 found in the intestine of two teleost fish species, *Barbatula barbatula* (L.) and *Cottus gobio* L.

**Results:**

We characterized 27 isolates of adult and larval parasites. Based on newly obtained *28S* and ITS1-*5.8S*-ITS2 rDNA sequences, new intermediate and final hosts were ascertained, and life-cycles clarified for some allocreadiids. New knowledge on the diversity and phylogenetic relationships of European *Crepidostomum* spp. was gained. The validity of *C. oschmarini* was verified based on comparative sequence analysis. Ophthalmoxiphidiocercariae of *C. oschmarini* were recorded in sphaeriid bivalves *Pisidium* (*Euglesa*) *casertanum* (Poli)*.* Additionally, morphological differences between gravid specimens of *C. oschmarini* and other related species were observed.

**Conclusions:**

Species of the Allocreadiidae parasitizing fishes in Europe are distributed among two monophyletic genera, *Allocreadium* and *Bunodera*, and two paraphyletic *Crepidostomum* clades. A complex of *Crepidostomum metoecus* (syn. *C. nemachilus)*, *C. oschmarini* and *Crepidostomum* sp. 2 clustered in one clade, and a complex of *C. farionis*, *Crepidostomum* sp. 1 and, probably, *C. wikgreni* in the other. Molecular data indicated that *C. oschmarini* and *Crepidostomum* sp. 2 presumably have a wide geographical distribution in Europe. The new data provided evidence that *Crepidostomum* is a more diverse genus than can be judged from morphological data and host switching in this genus may occur independently of fish-host phylogeny.

## Background

Trematodes of the genus *Crepidostomum* Braun, 1900 are common parasites in the intestine of freshwater teleosts in the Holarctic [1, 2]. Including many nominal species, the taxonomy of this genus still lacks clarity and the actual diversity and validity of some species is still questioned. Although most taxonomic decisions have been made based on adult morphology, it should be noted that a number of species are morphologically very similar and there exist only a few morphological features useful for distinguishing species [3, 4]. *Crepidostomum farionis* (Müller, 1784) and *C. metoecus* Braun, 1900 are among the most common and widely distributed freshwater parasites of salmonid fishes in Europe. Occasionally they are also found in *Cottus* spp. (Cottidae), *Barbatula barbatula* (L.) (Nemacheilidae) and some other fishes [5–7]. Reliable features for differentiating these two species were obtained only in the second half of the 20th century (see [7]) and formerly *C. metoecus* was frequently mistaken for *C. farionis*. Additionally, both species have been known by numerous synonyms (see [1, 8]). Prior to the present study, *C. farionis* and *C. metoecus* were the only representatives of the genus to have been recorded in Lithuania and neighboring regions [6, 9].

*Crepidostomum oschmarini* Zhokhov & Pugacheva, 1998 was described from the stone loach *Barbatula barbatula* (as *Nemacheilus barbatulus*) (Nemacheilidae) from the small Sutki River in the upper Volga River basin, Russia [10]. Later, the validity of this species was questioned based on a comparative study of the morphological variability of *Crepidostomum* spp., and *C. oschmarini* was synonymized with *C. metoecus* [11]. However, the definitive hosts of *C. metoecus* (salmonid fishes), have never been found in the Sutki River and the exact taxonomic status of *C. oschmarini* remained unresolved. Here, we re-visit the taxonomic status of *C. oshmarini* based on material from a new host, the European bullhead *Cottus gobio* (Cottidae, Scorpaeniformes), collected in the Il’d River, Russia. The main purpose of this study was to gain new knowledge on the diversity and phylogenetic relationships of European *Crepidostomum* spp. and to determine whether *C. oschmarini* and *C. metoecus* are distinct or synonymous by comparing the *5.8S*-ITS2 rDNA cluster and partial *28S* rDNA gene sequences, as well as to investigate the phylogenetic relationships of *C. oschmarini* within the Allocreadiidae. In addition to the molecular evidence provided, a detailed morphological study of *C. oschmarini* based on light and scanning electron microscopy (SEM) was accomplished. Despite the small number of studies on SEM morphology of *Crepidostomum* spp. it has been demonstrated that some surface features, such as the distribution of sensory endings, may provide additional specific characters for their identification [1, 7, 12, 13].

Life-cycle data and larval characters are equal in importance to adult characters for resolving some difficulties in taxonomy [1, 14]. Unfortunately, data for cercariae are lacking or incomplete for most ‘recognized’ *Crepidostomum* species [1]. Bivalves rather than gastropods are utilized as first intermediate hosts [2]. Known allocreadiid cercariae belong to the ophthalmoxiphidiocercariae type (i.e. with eye-spots and stylet) and develop in rediae [2]. The development of *Crepidostomum farionis* was elucidated by Brown [15]. Larval stages of *C. metoecus* were studied and described by Stenko [16] in Crimea (River Burulcha). The sphaeriid clam *Pisidium* (*Euglesa*) *casertanum* (Poli) was recorded as the first intermediate host, while larvae of the ephemeropteran *Ameletus* sp. served as the second intermediate host in experimental infection. Meanwhile, Awachie [17] found that the gastropod *Radix peregra* (O. F. Müller) (as *Lymnaea peregra*) serves as the first intermediate host for *C. metoecus* in North Wales and cercariae encysted in the amphipod *Gammarus pulex* (L.) as the second intermediate host (cercariae of allocreadiids encyst in aquatic arthropods). Due to this discrepancy between the two studies, it is likely that the authors were in fact dealing with different species.

The identification of intramolluscan stages of trematodes using morphological characters alone is difficult given their overall body plasticity and small size in relation to their complexity. The descriptions of cercariae of many related species render them morphologically indistinguishable. With molecular genetic methods having become standard practice for parasite identification, molecular data have become essential for matching different stages of digenean life-cycles. However, not a single life-cycle of a *Crepidostomum* species has been proven by molecular methods and only recently has a molecular study on trematodes in a sub-Arctic lake provided some molecular data on the diversity of developmental stages of some *Crepidostomum* spp. and their hosts in Europe [18].

During a parasite study of sphaeriid bivalves collected from different populations in Lithuania, Crimea and Norway, we found clams naturally infected with rediae and ophthalmoxiphidiocercariae consistent with the diagnosis and descriptions for allocreadiid cercariae. In the present study, rDNA markers of larval and adult allocreadiid stages were obtained and compared to known rDNA markers available for allocreadiid trematodes with the aim to clarify life-cycles, host specificity and phylogenetic relationships.

## Methods

Adult specimens of *C. oschmarini* were recovered from the intestine of *B. barbatula* and *C. gobio*. The fish hosts were caught in the Il’d River in the upper Volga River basin, Russia. Specimens of a few other adult allocreadiids, i.e. *Allocreadium isoporum* (Looss, 1894), *Bunodera luciopercae* (Müller, 1776) and *Crepidostomum* sp. 1 *sensu* Soldánová et al. (2017) [18], were recovered from fish hosts in Lithuania and Norway. Naturally infected sphaeriid clams were collected from different freshwater bodies in Lithuania, Norway and Crimea using hand-nets. The developmental stages of the allocreadiid species used in this study, their hosts, their sampling locality, and the GenBank accession numbers for the corresponding sequences, are presented in the Table 1.

**Table 1:** Species subjected to molecular phylogenetic analysis with information for hosts, localities and GenBank accession numbers

Species	Host	Locality	GenBank ID^b^ [Reference]	
			28S	ITS2
*Allocreadium* sp.^a^ (*= Crepidostomum* sp.)	*Sphaerium corneum*	Ukraine: River Belka, Dnieper River basin	GU462121 [44]	FJ874919 [44]
*Allocreadium* sp.^a^	*Pisidium amnicum*	Russia: River Tvertsa, upper Volga River basin		FJ874923 [44]
*Allocreadium gotoi*	*Misgurnus anguillicaudatus*	Japan: Nagano, Iiyama, Midori	LC215274 [61]	
*Allocreadium isoporum*	*Alburnus alburnus*	Russia: Lake Oster, Karelia	GU462125, GU462126 [44]	FJ874921 [44]
*Allocreadium isoporum*	*Barbatula barbatula*	Russia: River Il’d, upper Volga River basin	**MH143102**	**MH143096**
*Allocreadium lobatum*	*Semotilus corporalis*	USA: Moosehead Lake, Maine	EF032693 [62]	
*Allocreadium neotenicum*	*Hydroporus rufifrons*	United Kingdom: Lake District, Cumbria	JX977132 [43]	
*Allocreadium neotenicum*	*Oreodytes sanmarkii*	Norway: Lake Takvatn	KY513133 [18]	
*Allocreadium neotenicum* ^a^	*Pisidium casertanum*	Ukraine: River Burulcha, Crimea	**MH143103**	**MH143075**
*Allocreadium neotenicum* ^a^	*P. casertanum*	Norway: Lake Takvatn	**MH143104**	**MH143076**
*Allocreadium neotenicum* ^a^	*Pisidium* sp.	Norway: Lake Nordersjoen	**MH143105**	**MH143077**
*Auriculostoma* sp.	*Astyanax mexicanus*	Mexico: Filipinas, Veracruz		KF631425, KF631426 [63]
*Auriculostoma astyanace*	*Astyanax aeneus*	Costa Rica: Tempisquito River, Guanacaste	HQ833707 [64]	
*Auriculostoma lobata*	*Brycon guatemalensis*	Mexico: Mangal Lagoon, Tabasco	KX954172 [51]	
*Bunodera* sp.	*Perca flavescens*	USA: Steamboat Lake	HQ833704 [64]	
*Bunodera acerinae*	*Gymnocephalus cernuus*	Russia: Lake Segozero, Karelia	GU462114 [44]	FJ874914 [44]
*Bunodera acerinae* ^a^	*P. amnicum*	Russia: River Tvertsa, upper Volga River basin	GU462112, GU462113, GU462122 [44]	FJ874911 [44]
*Bunodera luciopercae*	*Perca fluviatilis*	Lithuania: Curonian Lagoon	**MH143101**	**MH143097**
*Bunodera luciopercae*	*P. fluviatilis*	Russia: Lake Segozero, Karelia	GU462115 [44]	FJ874917 [44]
*Bunodera luciopercae*	*P. fluviatilis*	Russia: River Tvertsa, upper Volga River basin	GU462123 [44]	FJ874918 [44]
*Bunodera luciopercae* ^a^	*Sphaerium rivicola*	Lithuania: dammed up River Nemunas near Kaunas	GU462116 [44]	FJ874916 [44]
*Bunodera luciopercae* ^a^	*S. rivicola*	Ukraine: River Teterev	GU462111 [44]	FJ874915 [44]
*Cercariaeum crassum* ^a^	*P. amnicum*	Lithuania: River Ūla	GU462120 [65]	JF261148 [65]
*Crepidostomum* sp. 1^a^	*Sphaerium* sp.	Norway: Lake Takvatn	KY513149 [18]	
*Crepidostomum* sp. 1^a^	*Siphlonurus lacustris*	Norway: Lake Takvatn	KY513150 [18]	
*Crepidostomum* sp. 1	*Salmo trutta*	Norway: Lake Sagelvvatn	**MH143111, MH143112**	**MH143080, MH143082**
*Crepidostomum* sp. 1^a^	*P. casertanum*	Norway: Lake Sagelvvatn	**MH143113, MH143114**	**MH143078, MH143081, MH143086**
*Crepidostomum* sp. 1^a^	*Pisidium* sp.	Norway: Lake Sagelvvatn	**MH143107, MH143108**	**MH143084, MH143085**
*Crepidostomum* sp. 1^a^	*Sphaerium nitidum*	Norway: Lake Kykkelvatn	**MH143106, MH143109, MH143110**	**MH143079, MH143083**
*Crepidostomum* sp. 2^a^	*P. casertanum*	Ukraine: River Burulcha, Crimea	**MH143117, MH143118, MH143119**	**MH143098, MH143099, MH143100**
*Crepidostomum* sp. 2^a^	*P. casertanum*	Norway: Lake Sagelvvatn	**MH143115, MH143116**	**MH143087, MH143088, MH143089**
*Crepidostomum* sp. 2	*S. trutta*	Norway: Lake Takvatn	KY513154 [18]	
*Crepidostomum* sp. 2	*S. lacustris*	Norway: Lake Takvatn	KY513151 [18]	
*Crepidostomum* sp. 2	*Diura bicaudata*	Norway: Lake Takvatn	KY513152 [18]	
*Crepidostomum affine*	*Hiodon tergisus*	USA: Pearl River, Mississippi	KF250358 [4]	
*Crepidostomum affine*	*Aplodinotus grunniens*	USA: Pearl River, Mississippi		KF356363 [4]
*Acrolichanus* (= *Crepidostomum*) *auriculatum*	*Acipenser schrenkii*	Russian Far East	FR821371 [30]	
*Crepidostomum auritum*	*Aplodinotus grunniens*	USA: Pearl River, Mississippi	KF250357 [4]	KF356373 [4]
*Crepidostomum cornutum*	*Lepomis gulosus*	USA: Pascagoula River, Mississippi	EF032695 [62]	KF356374 [4]
*Crepidostomum farionis* ^a^	*P. casertanum*	Norway: Lake Takvatn	KY513139 [18]	
*Crepidostomum farionis*	*Pisidium* sp.	Norway: Lake Takvatn	KY513136 [18]	
*Crepidostomum farionis*	*Oncorhynchus masou*	Russian Far East	FR821399, FR821402 [30]	
*Crepidostomum illinoiense*	*Hiodon alosoides*	USA: Red Lake River,Minnesota	KF356372 [4]	KF356364 [4]
*Crepidostomum metoecus*	*Salvelinus leucomaensis*	Russian Far East	FR821405, FR821406 [30]	
*Crepidostomum metoecus* (= *Crepidostomum nemachilus*)	*Barbatula toni*	Russian Far East	FR821408, FR821409 [30]	
*Crepidostomum metoecus*	*S. trutta*	Norway: Lake Takvatn	KY513148 [18]	
*Crepidostomum metoecus*	*P. casertanum*	Norway: Lake Takvatn	KY513140 [18]	
*Crepidostomum metoecus*	*Gammarus lacustris*	Norway: Lake Takvatn	KY513141 [18]	
*Crepidostomum oshmarini*	*B. barbatula*	Russia: River Il’d, upper Volga River basin	**MH159990, MH159992**	**MH143094, MH143095**
*Crepidostomum oshmarini*	*Cottus gobio*	Russia: River Il’d, upper Volga River basin	**MH159989, MH159991**	**MH143090, MH143091**
*Crepidostomum oshmarini* ^a^	*P. casertanum*	Lithuania: River Nedzingė	**MH159993, MH159994**	**MH143092, MH143093**
*Creptotrema funduli*	*Fundulus notatus*	USA: Mississippi, Biloxi River, Harrison County	JQ425256 [66]	
*Creptotrematina aguirrepequenoi*	*A. aeneus*	Costa Rica: Rio Tempisquito, Guanacaste	HQ833708 [64]	
*Phyllodistomum folium*	*Gymnocephalus cernuus*	Lithuania: Curonian Lagoon	KX957729 [46]	KY307885 [46]
*Phyllodistomum angulatum*	*Sander lucioperca*	Russia: Rybinsk water reservoir on the Volga river	KX957735 [46]	KJ740511 [46]
*Phyllodistomum macrocotyle* ^a^	*Dreissena polymorpha*	Belarus: Lake Lepelskoe	AY288828 [67]	AY288831 [67]

^a^Sequences from larval stages

^b^Sequences generated in the present study are indicated in bold

Adult trematodes were collected live from freshly killed fish. For molecular studies, the worms were rinsed in saline before being stored at 4 °C in 96% ethanol. Subsamples of the material for the morphological studies were fixed live in hot 10% buffered formalin. A total of 25 adult and gravid specimens of *C. oschmarini* (15 specimens from *B. barbatula* and 10 from *C. gobio*) were used for light microscopy examination. All measurements are in micrometres and are given as the range followed by the mean in parentheses.

For scanning electron microscopy, 19 live specimens of *C. oschmarini* from *B. barbatula* were fixed in 2.5% glutaraldehyde in 0.1 M phosphate buffer (pH 7.3) for 15 days at 4 °C. After washing in phosphate buffer, fixed worms were dehydrated though a graded ethanol series and acetone. They were then critical-point dried with liquid CO_2_ and mounted on stubs, sputter-coated with gold-palladium and examined using a JEOL JSM 6510LV scanning electron microscope (SEM) operating at 30 kV.

Genomic DNA was extracted from individual ethanol-fixed specimens following the protocol of Stunžėnas et al. [19] with a slight modification described in Petkevičiūtė et al. [20]. DNA fragments spanning the 3' end of the *5.8S* rRNA gene, the complete internal transcribed spacer 2 region (ITS2) and a small section at the 5' end of the *28S* gene were amplified using universal primers for flatworms, the forward primer 3S (5'-CGG TGG ATC ACT CGG CTC GTG-3') [21] and the reverse primer ITS2.2 (5'-CCT GGT TAG TTT CTT TTC CTC CGC-3') [22]. Using a new primer pair designed for species of the Allocreadiidae, the end of the internal transcribed spacer 1 (ITS1), the complete *5.8S* rDNA and ITS2, also a small section at the 5' end of the *28S* gene were amplified using the forward primer AlJe-F (5'-GTC TGG CTT GGC AGT TCT A-3') and the reverse primer AlJe-R (5'-CTG CCC AAT TTG ACC AAG C-3'). A fragment at the 5' end of the *28S* rRNA gene was amplified using the forward primers Digl2 (5'-AAG CAT ATC ACT AAG CGG-3') or ZX-1 (5'-ACC CGC TGA ATT TAA GCA TAT-3') [23] and the reverse primers L0 (5'-GCT ATC CTG AG (AG) GAA ACT TCG-3') [24] or 1500R (5'-GCT ATC CTG AGG GAA ACT TCG-3') [25, 26]. Amplification protocols are as described in Petkevičiūtė et al. [20]. The amplification protocol for the newly designed primers AlJe-F and AlJe-R is identical as for the primer pair Digl2-L0. PCR products were purified and sequenced in both directions at BaseClear B.V. (Leiden, Netherlands) using the PCR primers. Contiguous sequences were assembled using Sequencher 4.7 software (Gene Codes Corporation, Ann Arbor, USA). Sequences generated in this study have been deposited in the GenBank database (see accession numbers in Table 1).

Additional rDNA sequences for species of the Allocreadiidae and outgroup taxa (Table 1) were downloaded from GenBank and included in pairwise sequence comparisons and phylogenetic analyses. For phylogenetic analyses, the sequences were aligned using multiple sequence alignment software MAFFT version 7 [27] with iterative refinement method of G-INS-i. The best-fit model of sequence evolution for phylogenetic analysis was estimated using jModeltest v.0.1.1 software [28]. Maximum likelihood (ML) phylogenetic trees were obtained and analyzed using MEGA v.6 [29]. Branch support was estimated by bootstrap analyses with 1000 pseudoreplicates. The ML trees were obtained using the general time reversible model with a gamma distribution rate and a proportion of invariant sites (GTR + G + I) for both the ITS2 and the *28S* gene datasets. Gamma shape and the number of invariant sites were estimated from the data. Parsimony analysis based on subtree pruning and regrafting (SPR) was used with default parsimony settings. If two or more sequences belonged to one species, they were collapsed into one branch, except those newly obtained in this study*.* Estimates of mean evolutionary divergence over sequence pairs within and between groups were calculated using MEGA v.6.

## Results

**Family Allocreadiidae Looss, 1902**

**Genus** ***Crepidostomum*** **Braun, 1900**

ᅟ

***Crepidostomum oschmarini*** **Zhokhov & Pugacheva, 1998**

ᅟ

***Type-host*:** *Barbatula barbatula* (L.) (Cypriniformes: Nemacheilidae).

***Other host*:** *Cottus gobio* L. (Scorpaeniformes: Cottidae).

***Type-locality*:** River Sutki, Il’d River (the upper Volga River basin), Russia.

***Site in host*:** Intestine.

***First intermediate host*:** *Pisidium* (*Euglesa*) *casertanum* (Poli) (Veneroida: Sphaeriidae).

***Voucher material*:** Four voucher specimens ex *C. gobio* on 2 slides [No. 1/9(6–7)] and 6 voucher specimens ex *B. barbatula* on 2 slides [(No. 1/9(10–11)] were deposited in the Parasite Collection of the Institute for Biology of Inland Waters RAS, Russia.

***Representative DNA sequences*:** ITS2 rDNA (MH143090-MH143095); 28S rDNA (MH159989-MH159994) (see also Table 1).

### Description

**Fig. 1:**
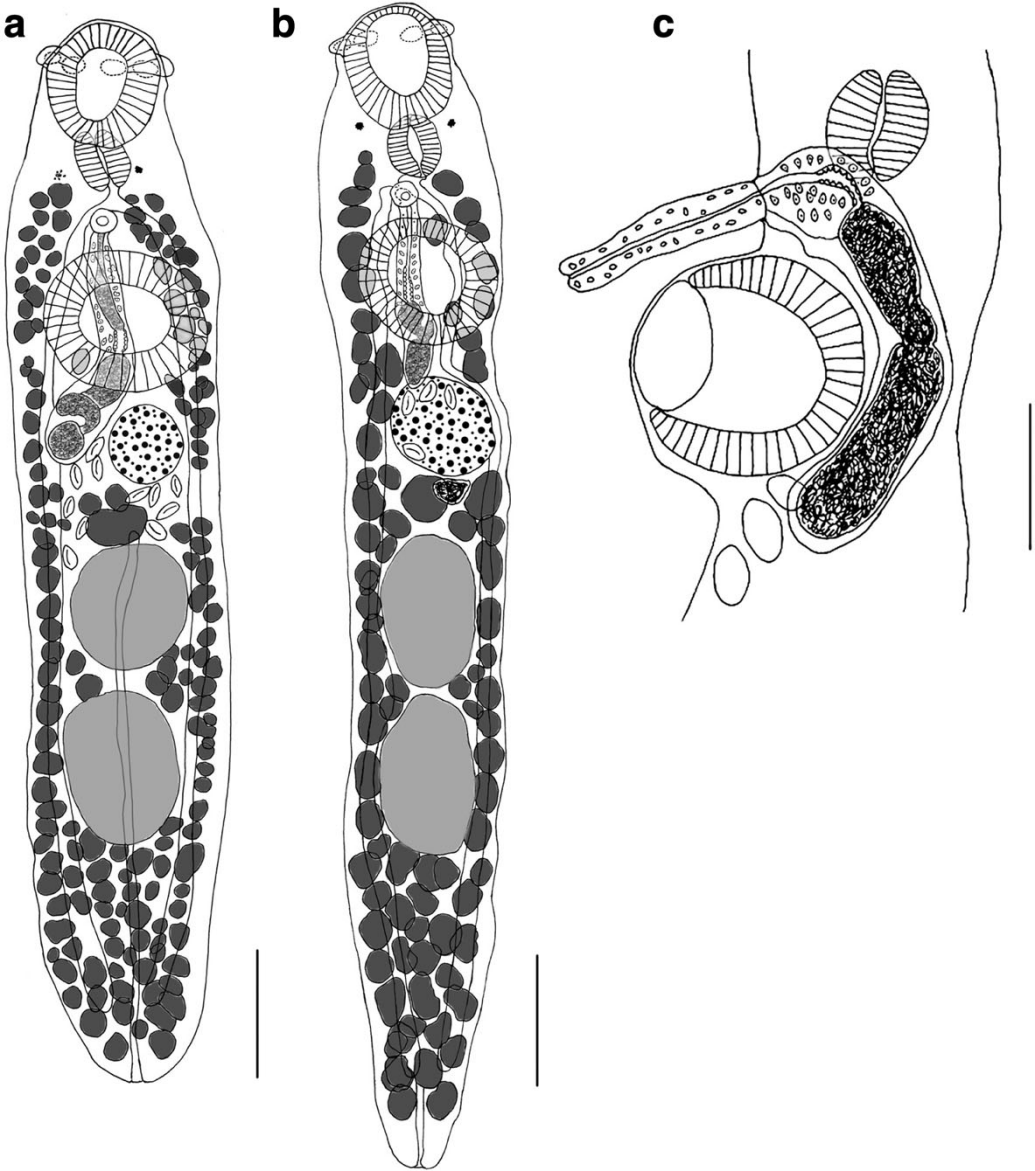
*Crepidostomum oschmarini*. **a** Whole-mount ventral view, ex *Barbatula barbatula*. **b** Whole-mount ventral view, ex *Cottus gobio.* **c** Terminal genitalia. *Scale-bars*: **a**, **b**, 200 μm; **c**, 100 μm

[Based on 25 ovigerous worms; Fig. 1, Table 2.] Body elongate-oval, spindle-shaped, only slightly dorsoventrally flattened, with bluntly rounded extremities, 1332–1872 (1561) long, with maximum width at level of ventral sucker, 225–396 (284) (Fig. 1a, b). Body width to body length ratio 1:4.4–8.2 (5.8); forebody 270–369 (320) long; hindbody 909–1458 (1229) long, forebody to hindbody length ratio 1:3–4.5 (1:3.9). Tegument smooth. Eye-spot pigment present in all specimens, usually solid, rarely dispersed. Oral sucker ventro-terminal, 136–222 × 143–210 (180 × 168), provided with 6 muscular lobes arranged in ventro-lateral, dorso-median and dorso-lateral pairs; lobes approximately equal in size and well-separated at their bases. Pre-oral lobe very short, 22–44 (29). Ventral sucker round, scyphoid, semi-embedded, almost equal in size to oral sucker, 163–234 × 156–288 (191 × 197); sucker width ratio 1–1.6 (1.3). Prepharynx short; pharynx muscular, elongate-oval, 55–101 × 68–92 (77 × 76); oesophagus short, 15–44 (26); intestinal bifurcation immediately posterior to pharynx, at approximately mid-way between suckers; caeca long, terminating blindly near posterior extremity of body.

**Table 2:** Measurements (in μm) of *Crepidostomum oschmarini* from *Barbatula barbatula* (*n* = 15) and *Cottus gobio* (*n* = 10)

Host species	*Barbatula barbatula*		*Cottus gobio*	
Variable	Range	Mean	Range	Mean
Body length	1332–1800	1432	1476–1872	1652
Maximum body width	264–396	309	225–300	263
Body width/length	4.4–8.2	5.1	4.9–8	6.4
Forebody length	270–342	305	297–369	334
Hindbody length	909–1300	1150	1170–1458	1307
Hindbody/forebody length	3–4.3	3.8	3.6–4.5	4
Pre-oral lobe length	22–66	44	22–44	29
Oral sucker length	136–189	156	180–222	201
Oral sucker width	143–185	154	144–210	180
Muscular lobes length	40–46	43	46–68	57
Muscular lobes width	22–24	23	22–35	29
Pharynx length	55–90	71	68–101	83
Pharynx width	68–77	73	68–92	79
Oesophagus length	26–44	33	15–26	20
Ventral sucker length	163–222	184	165–234	197
Ventral sucker width	156–288	203	165–228	192
Ovary length	92–136	113	128–176	155
Ovary width	99–139	112	121–163	138
Seminal receptacle length	55–59	57	46–82	57
Seminal receptacle width	44–73	59	55–99	74
Vitelline reservoir length	59–92	72	55–82	70
Vitelline reservoir width	79–99	87	57–110	76
Anterior testis length	158–233	183	216–246	235
Anterior testis width	121–209	156	130–198	170
Posterior testis length	176–242	200	222–330	262
Posterior testis width	132–220	163	132–210	178
Seminal vesicle length	66–209	146	66–143	109
Seminal vesicle width	44–88	70	30–66	47
Cirrus-sac length	244–420	332	242–450	322
Cirrus-sac width	48–82	71	48–77	60
Egg length	48–68	58	33–70	60
Egg width	26–35	32	29–37	34
Ventral sucker/oral sucker width	1–1.6	1.3	0.9–1.4	1.1
Oral sucker/pharynx length	1.9–2.6	2.2	2.2–2.8	2.5
No. of eggs	4–26	11	4–14	9

Testes 2, oval or globular, tandem, entire, contiguous or slightly separated; near middle of hindbody. Anterior testis 158–246 × 121–209 (210 × 164), smaller than posterior testis, latter 176–330 × 132–220 (233 × 171). Cirrus-sac elongate, well developed, 242–450 × 48–82 (327 × 66), with anterior end curved ventrally posterior to ventral sucker (Fig. 1c), extends posteriorly from level of intestinal bifurcation to ovarian region (up to posterior margin of ovary); contains coiled seminal vesicle, pars prostatica and ejaculatory duct. Seminal vesicle elongated, variable in size. Pars prostatica rectilinear, near middle of cirrus-sac; prostatic cells sparse, surround pars prostatica and extend throughout anterior half of cirrus-sac. Cirrus tubular, unarmed, 183–198 in length (*n* = 2). Genital atrium absent. Common genital pore median, between pharynx and ventral sucker, typically at level of intestinal bifurcation.

Ovary subspherical, entire, usually about equidistant between ventral sucker and anterior testis, dextrally or sinistrally submedian or in some specimens median, 92–176 × 99–163 (135 × 126). Proximal female genitalia not clearly observed. Seminal receptacle discerned in some specimens, 46–82 × 44–99 (57 × 66), rounded or somewhat transversely-elongate, immediately posterior to ovary. Uterus short, coiled between anterior testis and ventral sucker, overlapping ovary ventrally, only rarely extending slightly into testicular region; runs ventral to male duct; opens through common genital pore ventrally to male duct. Eggs not numerous, 4–26 (10 on average), operculate, thin-shelled, elongate-oval, 33–70 × 26–37 (59 × 33).

Vitellarium follicular, follicles in 2 lateral fields from level of posterior margin of pharynx to almost posterior extremity of body; fields sparsely confluent dorsally and ventrally in anterior caecal field, confluent dorsally and ventrally in post-testicular region, slightly overlapping testes, but not encroaching laterally between testes; no follicles present dorsal to ovary. Vitelline reservoir large, 55–92 × 57–110 (71 × 82), between ovary and anterior testis. Excretory vesicle tubular, elongate, I-shaped, reaches anterior margin of anterior testis. Excretory pore subterminal.

### Remarks

The specimens of *C. oschmarini* from *B. barbatula* are similar to those from *C. gobio* except in some morphological details that we do not consider to be of taxonomic importance. The worms from *C. gobio* have larger values for body length, body width to length ratio, oral sucker, muscular lobes on the oral sucker, pharynx, ovary, testes and eggs than the worms from *B. barbatula*. *Crepidostomum oschmarini* from *B. barbatula* also differs from the worms from *C. gobio* in the more rounded shape of testes.

Four species of the genus *Crepidostomum* (*C. metoecus*, *C. farionis*, *C. latum*, *C. wikgreni*) have been recorded from freshwater fishes in Europe. Here we do not consider *C. auriculatum* as this species is a specific parasite of sturgeons [1, 5] and appears to be much closer to *Bunodera* spp. than to its congeners in *28S* rDNA based molecular phylogenies ([18, 30], present study). The following species can be readily distinguished from *C. oschmarini*.

*Crepidostomum metoecus* differs in the larger size of the body and cirrus-sac, smaller hindbody to forebody length ratio of 1:2.2, longer oesophagus, larger number of eggs in uterus, 8–79 (mean 30) [1], and in the position of the genital pore (posterior to intestinal bifurcation) [1, 31]. The ovigerous worms from central Europe lack eye-spot pigment [31–34], the worms from Britain, Japan and USA have small scattered eye-spot pigment [1, 8, 35].

*Crepidostomum farionis* differs in the much larger size of the body, suckers, pharynx and eggs, and in having a much longer oesophagus and cirrus-sac, smaller forebody to hindbody length ratio of 1:2.5, as well as in the possession of separate genital pores which open anterior to the intestinal bifurcation [1, 36]. The ovigerous worms lack eye-spot pigment in the forebody [33, 34, 37–39], or eye-spot pigment is small and scattered [1, 8, 35, 40]. The uterus in *C. farionis* often extends into the testicular region (up to middle of anterior testes), the excretory vesicle is Y-shaped and eggs are numerous, up to 230 [34, 37, 38].

*Crepidostomum latum* Pigulewsky, 1931 has a wider body with a much shorter forebody and much larger size of eggs [41]. It differs from *C. oschmarini* by its shorter S-shaped cirrus-sac not reaching the posterior margin of the ventral sucker; shorter lateral fields of the vitellarium reaching anterior to the posterior margin of the ventral sucker; uterus extending into testicular region and lateral fields around the ventral sucker; testes which are almost equal in size.

*Crepidostomum wikgreni* is closest to *C. oschmarini* from which can be distinguished by its larger size of the body, pharynx, ventral sucker, ovary, testes, eggs, and in having a much longer oesophagus and shorter cirrus-sac and a specific microhabitat in the host (gall-bladder). Additionally, the ovigerous worms lack eye-spot pigment, the common genital pore is pre-bifurcal and the number of eggs in uterus is larger than in *C. oschmarini* (< 50 [42]).

The main morphological difference distinguishing *C oschmarini* from the species listed above is the eye-spot pigment in the forebody; this character was clearly present in all specimens. Furthermore, the very short oesophagus differentiates *C. oschmarini* from its congeners parasitizing salmonid fish hosts.

### Tegumental topography of *Crepidostomum oschmarini*

**Fig. 2:**
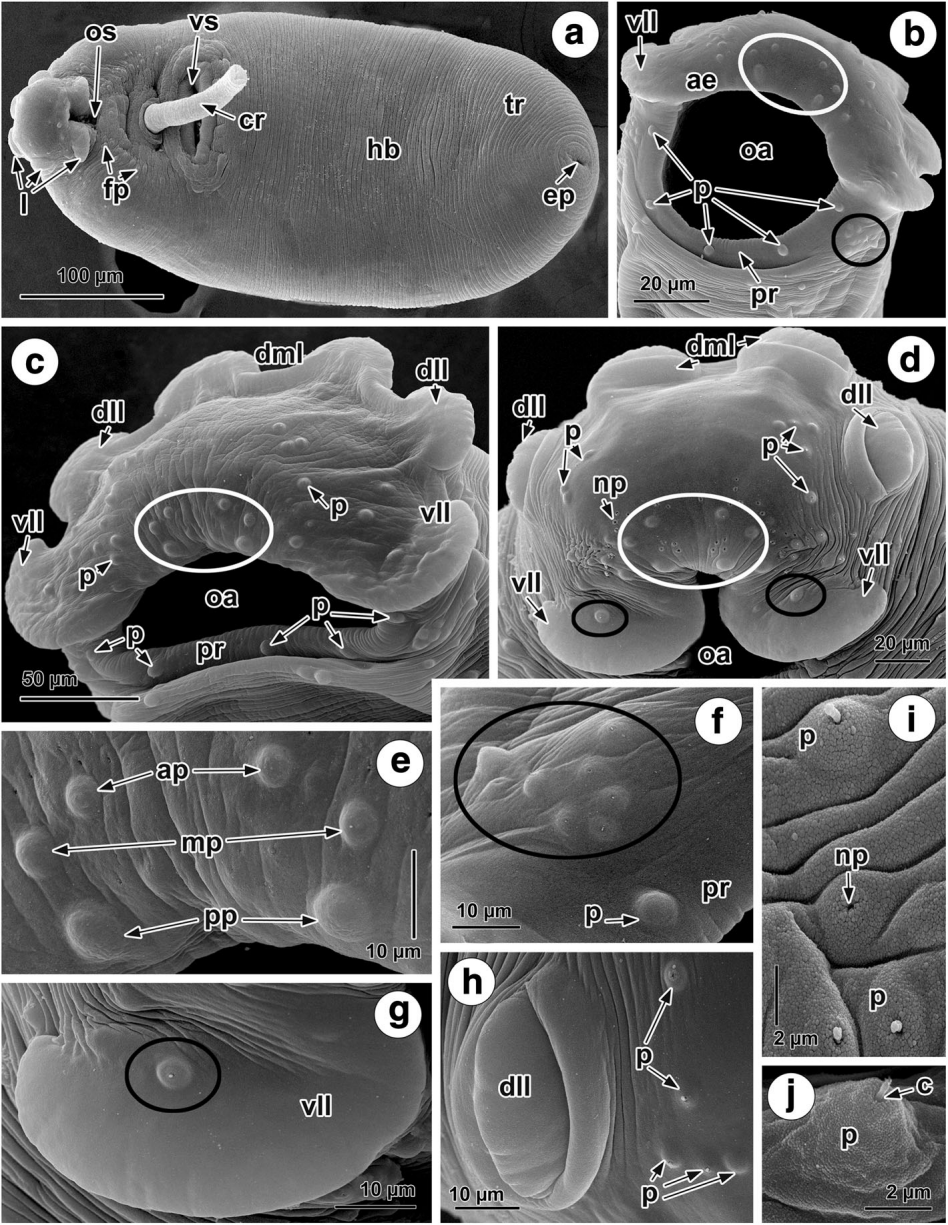
SEM micrographs of the surface topography of the anterior region of the body of *Crepidostomum oschmarini*. **a** Ventral view of mature worm. **b** The constant pattern of 5 papillae on the posterior rim of the oral sucker, 3 symmetrical pairs of papillae in the middle of the anterior rim (white circle) and a group of 8 papillae underneath the sucker rim (black circle). **c** 3 paired symmetrical papillae (white circle) and the distribution of irregular papillae on the anterior rim. **d** Interlobular field with marked (white circle) of 6 symmetrical papillae, irregular papillate and non-papillate sensory endings and single papilla on each ventro-lateral lobe (black circles). **e** Regular pattern of different sizes of the posteriormost, middle and anteriormost pairs of 6 symmetrical papillae on anterior rim. **f** Regular arrangement of 8 papillae ventro-lateral to the posterior sucker rim (black circle). **g** Single papilla (black circle) on the surface of a ventro-lateral lobe. **h** Papillae close to the base of a dorso-lateral lobe. **i** Ciliated papillate and non-papillate sensory endings on interlobular field. **j** Ciliated papilla. *Abbreviations*: ap, anteriormost papilla; c, cilium; cr, cirrus; dll, dorso-lateral lobe; dml, dorso-median lobe; fp, forebody papillae; hb, hindbody; ep, excretory pore; l, lobe; mp, middle papilla; np, non-papillate sensory ending; oa, oral aperture; os, oral sucker; p, ciliated papilla; pp, posteriormost papilla; pr, posterior rim of oral sucker; tr, transverse tegumental ridges; vll, ventro-lateral lobe; vs, ventral sucker

**Fig. 3:**
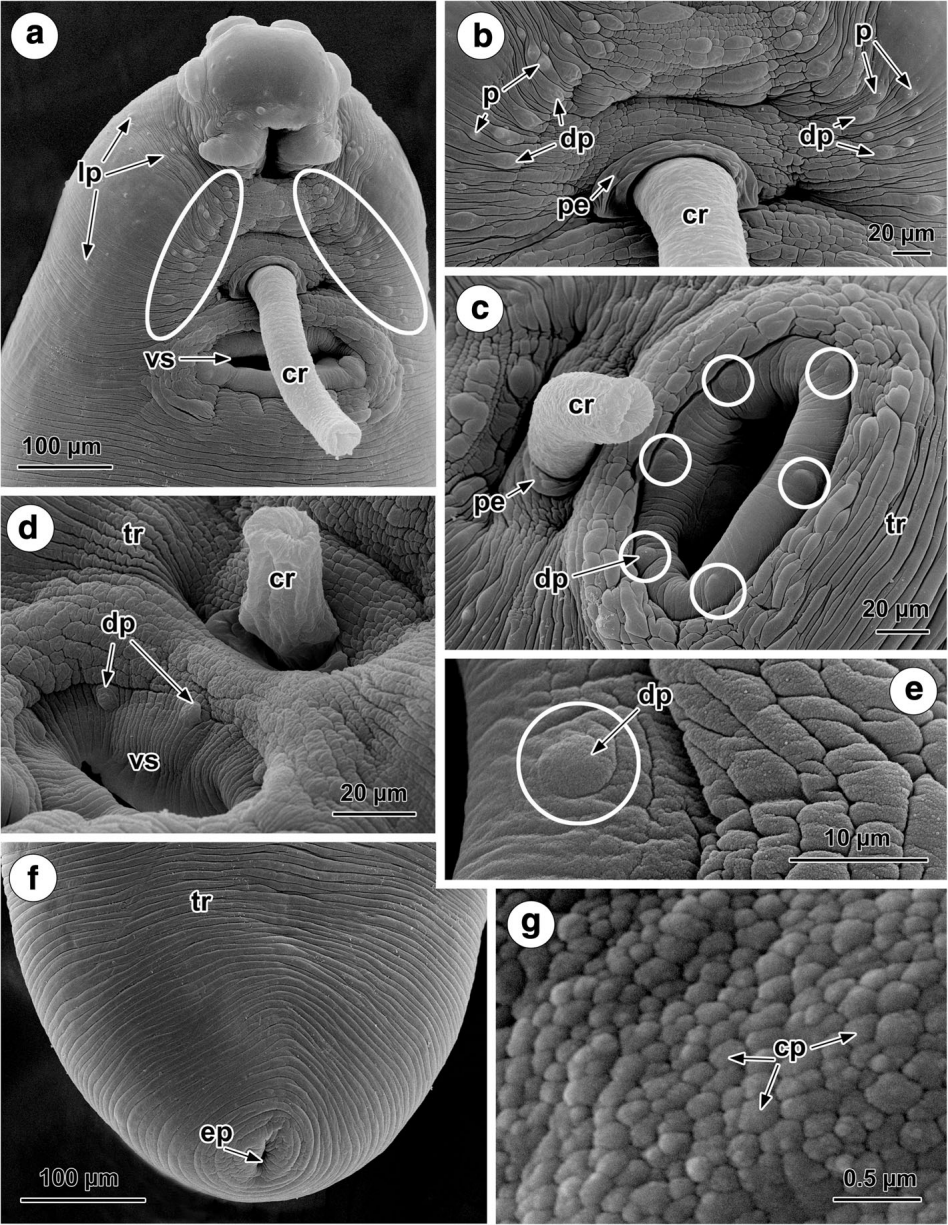
SEM micrographs of the surface topography of the forebody and hindbody of *Crepidostomum oschmarini.* **a** Two symmetrical longitudinal fields of papillae (white circles), the protruded cirrus and the ventral sucker on the forebody. **b** Ciliated and dome-shaped sensory endings in the two longitudinal fields of papillae. **c** Ventral sucker with 6 dome-shaped papillae on its rim (white circles). **d** Radially arranged surface corrugations on the rim of the ventral sucker and the dome-shaped papillae. **e** Dome-shaped papilla (white circle) on the ventral sucker rim. **f** Posterior extremity of the body with the terminal excretory pore. **g** Cobblestone-like protrusions of the body surface. *Abbreviations:* cp, cobblestone-like protrusion; cr, cirrus; dp, dome-shaped papilla; ep, excretory pore; lp, lateral papilla; p, ciliated papilla; pe, elevation around genital pore; tr, transverse tegumental ridges; vs, ventral sucker

Under SEM, the ventral and dorsal tegumental surface of *C. oschmarini* is unarmed and possesses transverse ridges (Figs. 2a and 3c, f). The presence of cobblestone-like protrusions on the body surface was apparent at a higher magnification (Fig. 3g). The anterior extremity of the body bears a ventro-terminal oral sucker provided with six protruding, muscular lobes (Fig. 2a, c, d). These lobes are arranged in three symmetrical pairs, ventro-lateral, dorso-lateral and dorso-median (Fig. 2c, d). The dorso-median and dorso-lateral lobes are approximately equal in size, whereas the ventro-lateral lobes are slightly wider. As they are continuous with the margin of the oral sucker, the anterior region of the ventro-lateral lobes form the anterior part of the oral sucker rim (Fig. 2b-c). There are numerous sensory endings in the form of so-called “papillae and minute sensory receptors” [1, 7, 12] on the oral sucker rim, around the oral sucker rim and along the interlobular field dorsally to the oral sucker (Fig. 2b-d). Five papillate sensory endings (*c.*6.5 μm in diameter) occur evenly spaced, about 40 μm apart from each other, and consistently associated with the posterior portion of the oral sucker rim (Fig. 2b, c). Underneath the ventro-lateral papilla of the oral sucker, groups of 8 papillae are visible on either side of the rim (Fig. 2c, f). In all of the specimens studied, there is a constant pattern of 3 pairs of symmetrical papillae, which vary in size, located at the middle of the anterior rim of the oral sucker (Fig. 2b-d, e). On each side of this symmetrical arrangement, the posteriormost papilla (*c.*7 μm in diameter) is situated about 5 μm from the middle papilla (*c.*4.5 μm in diameter), which, in turn, is situated about 4 μm from anteriormost papilla (*c.*3.5 μm in diameter) (Fig. 2e). The distance between posteriormost papillae on each side is 21 μm, between the middle papillae 32 μm and between anteriormost papillae 17 μm (Fig. 2e). An additional papilla (*c.*4.5 μm in diameter) is situated on the surface of each vento-lateral lobe (Fig. 2d, g). Two kinds of sensory endings, papillate and non-papillate, are scattered irregularly on the interlobular field of the body (Fig. 2c, d). A group of non-papillate sensory endings occurs close to the 3 pairs of symmetrical papillae (Fig. 2d). Variability exists in the numbers and arrangement of additional papillae in this region (Fig. 2c, d). Not far from the margins of the dorso-lateral lobes, groups of 3–5 papillae are present (Fig. 2d, h); furthermore, between the ventro-lateral and dorso-lateral lobes, 6 papillae and 3–5 non-papillate sensory endings are apparent (Fig. 2d). Non-papillate sensory endings are also present close to the margins of the dorso-median lobes. All of the sensory endings (papillate and non-papillate) on the anterior body surface are ciliate receptors (Fig. 2i, j).

The ventral sucker is protruded ventrally. Its rim exhibits surface corrugations arranged radially (Fig. 3d) and bears six large dome-shaped papillae (*c.*5.5 μm in diameter) regularly distributed around the rim (Fig. 3c-e).

The common genital pore is median between the two suckers (Fig. 5a, c); no tegumental papillae were observed around the genital pore (Fig. 3b-d). The tegument around the genital pore may form a weak elevation at the base of the everted cirrus (Fig. 3b, c). The surface of the cirrus is smooth (Fig. 3d). Everted cirrus was observed, measuring between 65–160 μm in length at different degrees of evagination (Fig. 3a, c, d).

Papillae are present on the forebody surface but are more abundant ventrally; they exhibit a tendency for bilateral symmetry (Fig. 3a, b). A gathering of ciliated and dome-shaped papillate sensory endings, arranged in two longitudinal, symmetrical rows, occurs on the ventral surface between the oral and ventral suckers (Fig. 3b). There are also a few papillae present in ventro-lateral and dorso-lateral areas of the forebody (Fig. 3a). The hindbody lacks papillae (Figs. 2a, 3f). Situated at the posterior extremity of the body is the excretory pore (Fig. 3f).

### Molecular differentiation and phylogenetic analysis

Two genetically different *Crepidostomum* cercariae were collected from *P. casertanum* in Lake Sagelvvatn in Norway, corresponding to *Crepidostomum* sp. 1 and *Crepidostomum* sp. 2 *sensu* Soldánová et al. (2017) [18] recorded in Lake Takvatn, Norway. One of these isolates was genetically identical to the metacercariae of *Crepidostomum* sp. 2 ex mayfly *Siphlonurus lacustris* and to adult ex *Salmo trutta* [18]. In Crimea, *Crepidostomum* sp. cercariae were found in *P. casertanum* collected in the River Burulcha. This cercaria has been described previously as the larva of *C. metoecus* [16], although the divergence between these two species ranged between 0.6–0.7% (7–8 bp) in the alignment of the *28S* gene. The divergence between the Crimean *Crepidostomum* sp*.* and the sub-Artic *Crepidostomum* sp. 2 can be regarded as intraspecific, with 2–3 bp (0.2–0.3%) difference in the *28S* and only 1 bp (0.16%) in the *5.8S*-ITS2.

Adult *Crepidostomum* specimens obtained from *S. trutta* in Lake Sagelvvatn were genetically identical to samples of cercariae from *P. casertanum* and *Pisidium* sp. collected in this lake and from *Sphaerium nitidum* in Lake Kykkelvatn. The sequences of the *28S* rRNA gene obtained from all of these samples were identical to *Crepidostomum* sp. 1 samples collected from *Sphaerium* sp. and *S. lacustris* in Lake Takvatn [18]. Intraspecific variation was detected in Lake Sagelvvatn, but only a single nucleotide in the ITS2 was different in one specimen from *S. trutta* and in one isolate from *Pisidium* sp.; also this site was heterozygotic in one sample from *P. casertanum*, obviously as a result of hybridization of two genetically different lineages*.*

The newly generated rDNA sequences of *C. oschmarini* sampled from *B. barbatula* and *C. gobio* in Russia, and from *P. casertanum* in Lithuania were identical. The *28S* sequences were aligned with those of closely related species in an alignment of 1150 bp. The divergence between *C. oschmarini* and *C. metoecus* was 8 and 9 bp (0.7–0.8%), the divergence between *C. oschmarini* and the sub-Arctic *Crepidostomum* sp. 2 was 11 and 12 bp (0.96–1%). However, *C. oschmarini* was less different from the Crimean *Crepidostomum* sp. 2 (9 bp, 0.8%).

Some specimens of *P. casertanum*, collected in the River Burulcha and in Lake Takvatn, Norway, as well as *Pisidium* sp. from Lake Nordersjoen, were infected with *Allocreadium neotenicum.* All samples were genetically identical. In a *28S* alignment of 1150 bp, no nucleotide differences were detected between our samples and *A. neotenicum* collected from the dytiscid beetles *Oreodytes sanmarkii* and *Hydroporus rufifrons* in the sub-Arctic Lake Takvatn and the Lake District in Cumbria, UK, respectively [18, 43].

New rDNA sequences were obtained for *A. isoporum*, collected from *B. barbatula* in the upper Volga River basin, Russia*.* These sequences were identical to rDNA of *A. isoporum* collected from *Alburnus alburnus* in Lake Oster, Karelia, Russia [44], with one difference in a single nucleotide of the ITS2.

New rDNA sequences were obtained for *B. luciopercae*, collected from *Perca fluviatilis* in the Curonian Lagoon, Lithuania*.* The divergence between sequences from this specimen and sequences of *B. luciopercae* specimens from a previous study [44] was 2 and 3 bp in a *28S* alignment of 1067 bp and only 1 bp in the ITS2 alignment of 394 bp.

The newly obtained sequences and relevant allocreadiid sequences of ITS2 rDNA and partial *28S* rDNA from the GenBank database were used for phylogenetic analysis. Alignment of the ITS2 and partial *28S* data yielded 394 and 1050 characters for analysis, respectively.

**Fig 4:**
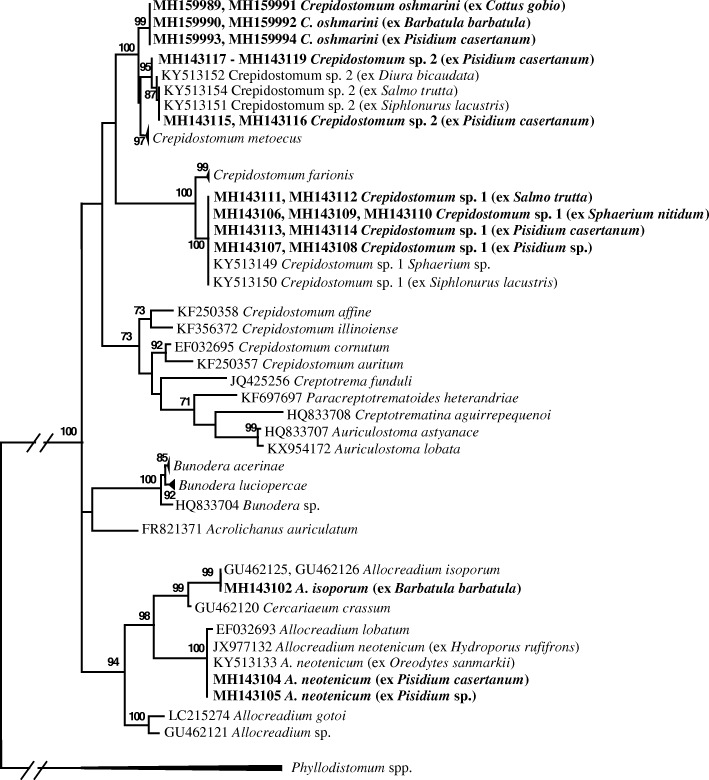
Phylogenetic tree based on Maximum Likelihood analysis of partial sequences of the *28S* nuclear rRNA gene. Bootstrap support values lower than 70% are not shown. GenBank accession numbers of sequences in collapsed clades are provided in Table 1. The species sequenced in this study are indicated in bold

**Fig. 5:**
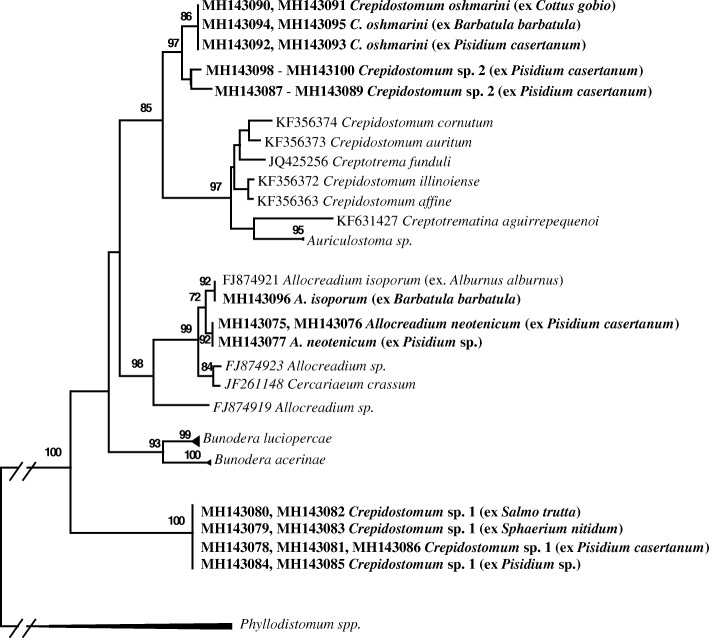
Phylogenetic tree based on maximum likelihood analysis of the ITS2 nuclear rDNA region. Bootstrap support values lower than 70% are not shown. GenBank accession numbers of sequences in collapsed clades are provided in Table 1. The species sequenced in this study are indicated in bold

Phylogenetic analyses of the ITS2 and *28S* datasets produced several strongly supported clades and some weakly or not supported clades in both phylogenetic trees (Figs. 4, 5). Adult *C. oschmarini* from two fish species, *B. barbatula* and *C. gobio*, and allocreadiid cercariae from the sphaeriid bivalve *P. casertanum*, formed a strongly supported monophyletic subclade (Figs. 4, 5). Sub-Arctic *Crepidostomum* sp. 2 together with Crimean *Crepidostomum* sp. formed the other subclade. These two subclades nested in to a well-supported monophyletic clade; in the *28S* tree *C. metoecus* is included into this clade. *Crepidostomum* sp. 1 formed the other monophyletic clade (Fig. 5); in the *28S* tree, together with *C. farionis* (Fig. 4). This clade was nested as sister to the clade formed by *C. oschmarini + Crepidostomum* sp. 2 + *C. metoecus* but the relationship was not supported (Fig. 4). Unfortunately, ITS2 data for *C. metoecus* and for *C. farionis* are not yet available. Nearctic *Crepidostomum* species nested into a separate monophyletic clade together with species from the allocreadiid genera *Auriculostoma* and *Creptotrematina* in the two trees and, additionally, with *Creptotrema* and *Paracreptotrematoides* in the *28S* tree*.* Species of *Allocreadium*, as well as species of *Bunodera*, nested into two separate monophyletic clades (Figs. 4, 5). However, the relationships among all these clades showed some differences in the different trees. In the *28S* rDNA tree, all these clades and the branch of *Acrolichanus auriculatum* were united into one strongly supported main clade, but did not form supported higher-level clades inside the main clade. The main difference between the ITS2 and *28S* rDNA trees was in the relationships of *Crepidostomum* species. In the ITS2 tree, the strongly supported clade of Nearctic *Crepidostomum* spp. and the clade of *C. oschmarini-Crepidostomum* sp. 2 nested into a well-supported higher-level clade (Fig. 5), while in the *28S* tree (Fig. 4), the clade of Nearctic *Crepidostomum* spp. was not strongly supported and its relationships with other clades of *Crepidostomum* was not supported.

## Discussion

The results of this study shed new light on the diversity of trematodes of the genus *Crepidostomum* in Europe. The existing genetic data was based on analysis of partial sequences of the *28S* rRNA gene of *Crepidostomum* species that use salmonid fishes as final hosts [18]. *Crepidostomum oschmarini* was found from two sympatric but phylogenetically distant fish host species, *B. barbatula* (Cypriniformes) and *C. gobio* (Scorpaeniformes). Although host switching is probably easier among related host species [45], host switches between unrelated hosts can also take place [46–48]. While *B. barbatula* and *C. gobio* are phylogenetically distant, their feeding habitats and food preferences are similar. Predominant preys are small benthic arthropods (insect larvae, i.e. ephemeropterans, plecopterans, trichopterans and crustaceans [49, 50]). It is likely that a feeding overlap produced by food items that are involved in the life-cycle of the parasite, resulted in the infection of both fish with the same *Crepidostomum* species.

Comparative sequence analysis in this study confirmed the link between the redial and cercarial isolates ex *P. casertanum* from the River Nedzingė, Lithuania, and the adult stages of *C. oschmarini* parasitizing *B. barbatula* and *C. gobio* from the River Il’d, Russia. No intraspecific variation was detected between these isolates, despite the considerable geographical distance (~ 1000 km) between the Lithuanian and Russian populations.

The level of differences found between the partial *28S* rDNA sequences of *C. oschmarini* and *C. metoecus* (0.6–0.7%) clearly demonstrates that these two forms are different species. The observed level of differences is similar to the levels of interspecific variability reported for allocreadiid digeneans [4, 18, 30, 44]. It is notable that occasionally relatively low interspecific genetic divergence can be discovered in related allocreadiid species; for instance, only 0.29% divergence was found between *Auriculostoma lobata* Hernández-Mena, Lynggaard, Mendoza-Garfias & Pérez-Ponce de León, 2016 and its sister species *A. astyanace* Scholz, Aguirre-Macedo & Choudhury, 2004 (Allocreadiidae) [51]. Despite the conservative nature of the *28S* rDNA gene region, it segregates well-supported subclades of *C. metoecus*, *Crepidostomum* sp. 2 and *C. oschmarini*, within a single clade (Fig. 4).

Recently, the existence of cryptic species was uncovered among *Crepidostomum* spp. infecting salmonid fishes and different first and second intermediate hosts in the sub-Arctic Lake Takvatn [18]. Hence, molecular results evidenced that species diversity in *Crepidostomum* is underestimated. Molecular data obtained from analysis of *28S* rDNA partial sequences disclosed two pairs of genetically closely related species, i.e. *C. farionis* - *Crepidostomum* sp. 1, and *C. metoecus*-*Crepidostomum* sp. 2. Considering that there are only five nominal European *Crepidostomum* species included into the Fauna Europaea database [52], i.e. *C. auriculatum* Wedl, 1858, *C. farionis*, *C. latum*, *C. metoecus* and *C. wikgreni* Gibson & Valtonen, 1988, the finding was surprising and shows that diversity in this genus and in this otherwise depauperate freshwater ecosystem is higher than was presumed. *Crepidostomum farionis* and *C. metoecus* were known as the only two species of this genus parasitizing salmonids in Europe [5, 34]. *Crepidostomum wikgreni* described from the gall-bladder and intestine of the whitefish *Coregonus acronicus* (Salmonidae) in Lake Yli-Kitka, northeast Finland, has never been recorded elsewhere and is regarded as an endemic form [42]. No sequence data are available for this species and it could not be included in molecular phylogenies. The gross morphology of *C. wikgreni* appeared very similar to *C. farionis* and it was suggested that *C. wikgreni* has probably evolved from *C. farionis* after deglaciation and since *c.*8400 BP when the waters of the Kitka Lake system were isolated [42]. These two species presumably represent closely related sister taxa.

*Crepidostomum nemachilus* was originally described from *Nemachilus barbatulus toni* (Nemacheilidae) on Sakhalin Island in the Russian Far East [53]. The genetic identity of *C. nemachilus* and *C. metoecus* was revealed in comparative analysis of *28S* sequences, but distinctions observed in morphology of the two species were regarded as an argument to refrain from deciding upon synonymy of the two taxa [30]. However, recent morphological reexamination of *C. nemachilus* from the type-host *Barbatula toni* (Dybowski) (syn. *Nemachilus toni*) showed that it is consistent with the specimens of *C. metoecus* including those found in *B. toni* in every essential feature [8]. Thus, both molecular and morphological findings demonstrated that *C. nemachilus* is a synonym of *C. metoecus*.

*Acrolichanus auriculatum* (syn. *Crepidostomum auriculatum*), a parasite of sturgeons, comprise a separate branch in the molecular *28S* rDNA phylogenetic tree, distantly related to other *Crepidostomum* spp. [18, 30]. Wedl [54] described this species as *Distoma auriculatum*. Since then, it has undergone many taxonomic revisions (see [1, 55]). Skwortzoff [56] conducted a comprehensive morphological analysis of the species based on a large amount of material collected from *Acipenser ruthenus* from the Volga River and Oka River, concluding that they should be assigned to the genus *Acrolichanus* Ward, 1917. Thus, there have been opposing opinions on the validity of *Acrolichanus*. Some authors, along with Hopkins [57], are of the opinion that this taxon is insufficiently distinct from *Crepidostomum* and must be placed within the latter genus. However, molecular data support the opinion of Skryabin & Koval [55] and Bykhovskaya-Pavlovskaya & Kulakova [5] that *A. auriculatum* is distinct enough from samples of the other Palaearctic and Nearctic *Crepidostomum* spp. to be assigned to another genus. In *28S* rDNA based phylogenies *A. auriculatum* appears to be much closer to *Bunodera* spp. than to *Crepidostomum* spp.

*Crepidostomum latum* is a little-known species described by Pigulewsky [41] based on only two specimens from the intestine of the rudd, *Scardinius erythrophthalmus* (L.) (Cyprinidae), in the River Sozh (in the upper course of the River Dnepr, Ukraine). The species has not been encountered since and its validity is questionable [5].

The *28S* rDNA based phylogenetic tree generated here agrees in general topology with recently published estimates of phylogeny for the Allocreadiidae [18, 58]. In these studies, the species of the genera *Allocreadium* and *Bunodera* formed two monophyletic clades. The different situation concerns the genus *Crepidostomum.* In the present analyses *C. oschmarini*, *C. metoecus* (syn. *C. nemachilus* Krotov, 1959) and *Crepidostomum* sp. 2 represented closely related sister taxa in the *28S* rDNA-based phylogeny. We refer to this clade as the *C. metoecus* complex. The second clade, including European *Crepidostomum* isolates was comprised of *C. farionis* and *Crepidostomum* sp. 1. Our sequences for isolates sampled from *Sphaerium nitidum*, *Pisidium* sp., *P. casertanum* and *S. trutta* from Norway matched the sequences of *Crepidostomum* sp. 1 of Soldánová et al. [18]. However, a monophyletic origin of the two *Crepidostomum* clades is not supported in the *28S* rDNA-based phylogeny. DNA sequences, unconstrained by function, as the internal transcribed spacer 2 (ITS2), usually experience higher rates of genetic change than encoding regions, as the *28S*. In the ITS2-based phylogeny, the *Crepidostomum* sp. 1 clade was distant from all other allocreadiid clades, but the *C. metoecus* complex and Nearctic *Crepidostomum* clade formed well-supported higher-level clade (Fig. 5). It is interesting that the Nearctic *Crepidostomum* clade combines some species of the other allocreadiid genera, i.e. *Auriculostoma* and *Creptotrematina* in the ITS2 tree, and even more genera comprise this clade in the *28S* tree*.* The results of the phylogenetic analyses led us to a presumption that at least two groups of *Crepidostomum* species are paraphyletic. There are more cases known when molecular analysis using the *28S* rDNA gene revealed paraphyly in a group of allocreadiid trematodes, conventionally regarded as a monophyletic assemblage. Hence, recently the genus *Paracreptotrema* Choudhury, Pérez-Ponce de León, Brooks & Daverdin, 2006 was shown to be a paraphyletic and two new genera were erected to accommodate the taxonomy with the results of molecular phylogeny [58].

The sequences of isolates of the intramolluscan stages from *P. casertanum* collected in the River Burulcha, Crimea, formed a robustly supported subclade with metacercarial and adult isolates of *Crepidostomum* sp. 2 *sensu* Soldánová et al. (2017) [18] from a sub-Arctic lake in Norway. This finding did not match our expectations, as the upper stream of the River Burulcha, Crimea, is the type-locality for the cercarial material used by Stenko [16] for experimental life-cycle studies on *C. metoecus*. Notably, the second intermediate hosts recorded in the study of Stenko [16] were nymphs of the mayfly (Ephemeroptera) and stonefly (Plecoptera). Nymphs of these insects were found infected with metacercariae of *Crepidostomum* sp. 2 in the molecular study of Soldánová et al. [18]. On the other hand, amphipods *Gammarus lacustris* were found to be the second intermediate host of *C. metoecus* in Lake Takvatn in Norway [18]. Based on these facts, we can assume that Stenko [16] was dealing with *Crepidostomum* sp. 2. Adult specimens of *Crepidostomum* sp. 2 were recorded in brown trout, *S. trutta*, in the molecular study of Soldánová et al. [18], and Stenko [16] noted that *S. trutta fario* was naturally infected with “*C. metoecus*” in the River Burulcha. Speciation patterns of parasites may be directly associated with their hosts, though in the case of parasites with complex life-cycles it is often less clear which host may have the most influence on parasite speciation. The life-cycle peculiarities of two closely related species, *C. metoecus* and *Crepidostomum* sp. 2, would suggest that the speciation was driven by factors associated with the second intermediate hosts, phylogenetically distant arthropods, while the first intermediate and definitive hosts are shared between these two trematode species. However, a more accurate knowledge of life-cycles is necessary to explain the pattern of cryptic diversity observed in *Crepidostomum* spp.

The molecular segregation of *C. oschmarini* and *C. metoecus* prompted us to compare these worms using SEM and to try to identify diagnostic morphological features for the species. The present SEM study of the surface morphology of *C. oschmarini* revealed both common and specific patterns in the number and arrangement of tegumental papillae as compared with other similarly studied species of *Crepidostomum* [1, 7, 12, 13].

The five large papillae detected on the posterior rim of the oral sucker of *C. oschmarini* are common to all of the *Crepidostomum* species examined to date and can be seen in SEM photos of *C. metoecus*, *C. farionis*, *C. illinoiense* Faust, 1918, *C. ictaluri* (Surber, 1928) and *C. cooperi* Hopkins, 1931, published by Caira [1], as well as in *C. farionis* and *C. metoecus* examined by Moravec [7] and Žd'árská & Nebesářová [13] and also in *C. opeongoensis* Caira, 1985 studied by Choudhury & Nelson [12]. Five characteristic larger papillae are also visible on the posterior rim of the oral sucker in other allocreadiid species, e.g. *Bunodera sacculata* Van Cleave & Mueller, 1932 and *B. mediovitellata* Tsimbaliuk & Roitman, 1966 studied by Caira [1].

The presence of six dome-shaped papillae on the rim of the ventral sucker revealed in *C. oschmarini* has also been observed in *C. metoecus* by Moravec [7] and in *C. opeongoensis* by Choudhury & Nelson [12]. However, no papillae were observed on the ventral sucker in *C. farionis* [7].

A consistent pattern in the sensory papillae arrangement was found to occur in the anterior body region and ventral forebody surface of all specimens of *C. oschmarini*. First, there are three paired, symmetrically distributed, differently-sized papillae situated in the centre of the anterior rim of the oral sucker. Such a pattern has not been reported for any of the other *Crepidostomum* species studied to date by SEM [1, 7, 12, 13]. In *С. metoecus*, a species most closely related to *C. oschmarini*, only two pairs of symmetrically arranged papillae are visible in the illustrations of Moravec [7] and Žd'árská & Nebesářová [13].

Secondly, a local concentration of eight papillae located ventro-laterally underneath the rim of the oral sucker is characteristic for *C. oschmarini*. It is worth noting, however, that the presence of a similar but smaller group of papillae can be seen in published SEM photos of some previously studied species, i.e. three papillae in *C. metoecus* and five papillae in *C. cooperi* [1].

Thirdly, a single large papilla is associated with each ventro-lateral lobe in *C. oschmarini*. In contrast, the presence of two papillae on the surface of each ventro-lateral lobe is apparent in the SEM photos of *C. metoecus* presented by Moravec [7].

Fourthly, the arrangement of ciliated and non-ciliated sensory endings in two longitudinal symmetrical rows on the ventro-median surface of the forebody was revealed in *C. oschmarini.* Judging from the available SEM data on the papillae distribution in *Crepidostomum* spp., two fields of “tegumental bosses” (non-ciliated sensory endings) are situated laterally along the forebody in *C. metoecus* [7] and, there are four pairs of papillae on the ventral forebody in *C. opeongoensis* [12].

The present SEM study clearly demonstrates the distinction between two sister taxa, *C. oschmarini* and *C. metoecus*, which have been shown, using our molecular data, to be closely related. This and previous SEM studies on the surface topography of species of *Crepidostomum* suggest that the arrangement of the sensory endings of adult specimens exhibit interspecific differences which represent useful additional taxonomic criteria for understanding this genus.

## Conclusions

According to available data, we suggest that two complexes of *Crepidostomum* species parasitize freshwater fishes in Europe. The *Crepidostomum metoecus* complex consists of *C. metoecus* (syn. *C. nemachilus)*, *C. oschmarini* and *Crepidostomum* sp. 2, while the *C. farionis* complex includes *C. farionis*, *Crepidostomum* sp. 1 and, probably, *C. wikgreni*. Morphological and molecular evidence together indicated the validity of *C. oshmarini* and provided clear criteria for its separation from *C. metoecus* and other congeneric species. The phylogenetic study supported that some *Crepidostomum* species are euryxenous, so host switching in this genus may occur independently of fish-host phylogeny. Our phylogenetic analyses confirm the prediction that there are large numbers of cryptic parasite species to be discovered [59] and reinforce the idea that trematodes are a much more diverse group than as is judged from morphological data and once again confirm the observation that studies based on comparison of nuclear DNA markers are more likely to uncover cryptic species among trematodes than other groups of helminths [60]. This study demonstrates the value of steadily adding relevant parasitological and sequence data to a growing database for allocreadiids as well as for any other group of trematodes. No matter which life-cycle stage has been obtained or from what hosts and geographical localities, from such specimens we will gain the framework needed to connect and clarify life-cycles and gain a more complete understanding of the existing diversity, host specificity and ecology of trematodes under consideration.

## Abbreviations

GTR + G + I: Gamma distribution of rates and a proportion of invariant sites
ITS2: Internal transcribed spacer 2
ML: Maximum likelihood
SEM: Scanning electron microscopy
SPR: Subtree pruning and regrafting
